# γδ T Lymphocytes in Asthma: a Complicated Picture

**DOI:** 10.1007/s00005-021-00608-7

**Published:** 2021-03-04

**Authors:** Michał K. Zarobkiewicz, Ewelina Wawryk-Gawda, Wioleta Kowalska, Mariola Janiszewska, Agnieszka Bojarska-Junak

**Affiliations:** 1 Department of Clinical Immunology, Medical University of Lublin, Chodźki 4a, 20-093 Lublin, Poland; 2 Department of Paediatric Pulmonology and Rheumatology, Medical University of Lublin, Lublin, Poland; 3 Department of Medical Informatics and Statistics With E-Learning Laboratory, Medical University of Lublin, Lublin, Poland

**Keywords:** γδ T lymphocytes, Asthma, γδ T subsets

## Abstract

A minor subset (approximately 5%) of peripheral T cells has their TCR build up from γ and δ chains instead of α and β—those are the γδ T lymphocytes. They can be functionally divided into subsets, e.g., Th1-, Th2-, Th9-, Th17-, Tfh-, and Treg-like γδ T cells. They share some specifics of both innate and adaptive immunity, and are capable of rapid response to a range of stimuli, including some viral and bacterial infections. Atopic diseases, including asthma, are one of major health-related problems of modern western societies. Asthma is one of the most common airway diseases, affecting people of all ages and having potential life-threatening consequences. In this paper, we review the current knowledge about the involvement of γδ T cells in the pathogenesis of asthma and its exacerbations. We summarize both the studies performed on human subjects as well as on the murine model of asthma. γδ T cells seem to be involved in the pathogenesis of asthma, different subsets probably perform opposite functions, e.g., symptom-exacerbating Vγ1 and symptom-suppressing Vγ4 in mice model of asthma.

## Introduction

**Fig. 1:**
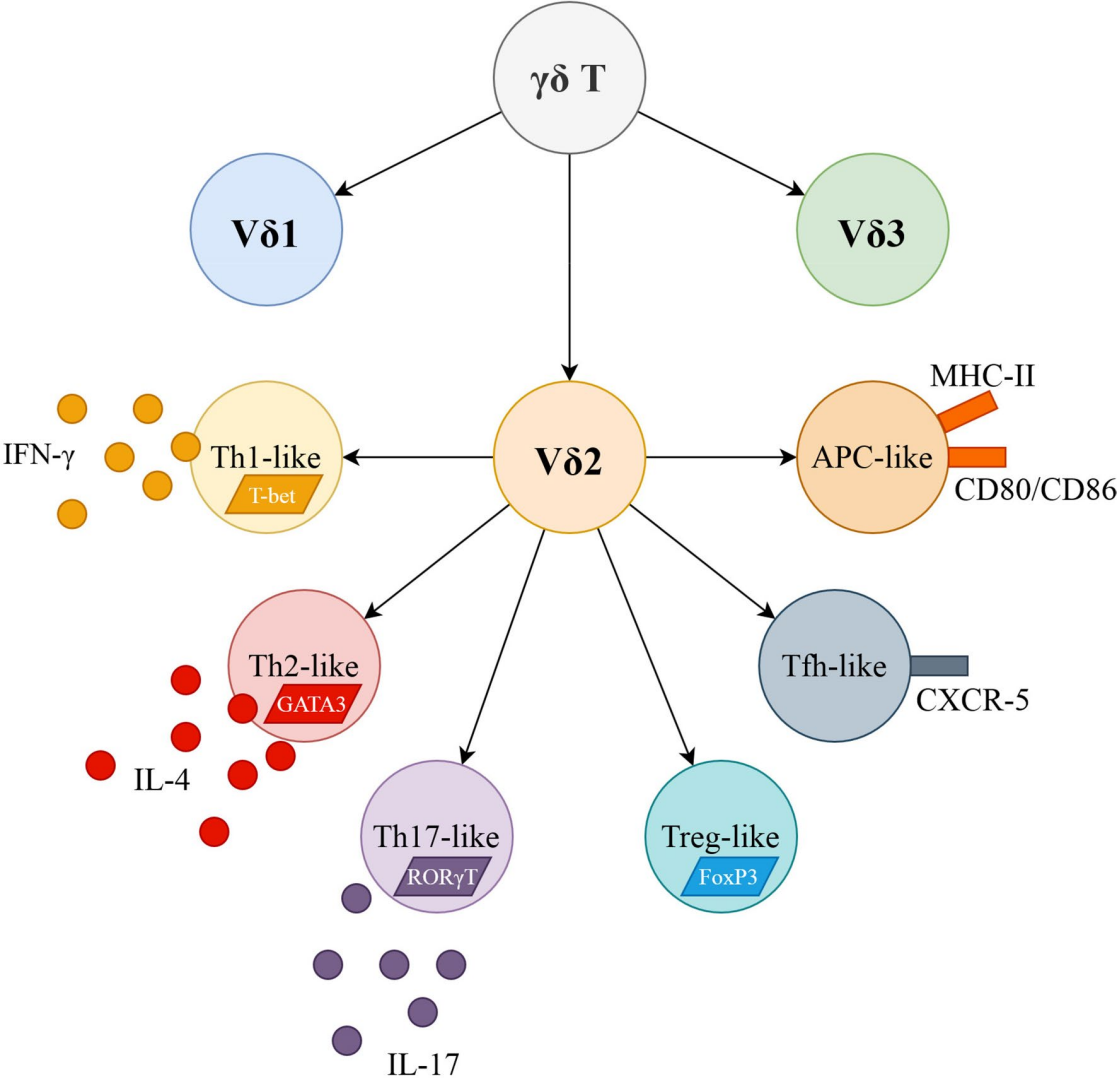
Major populations of human γδ T cells as proposed by Pang et al. (2012). Most important surface antigens, cytokines produced by them as well as their transcription factors are presented

Majority of T cells, frequently referred to as conventional T cells, express T cell receptor (TCR) comprised of α and β chains, while approximately 5% of peripheral T cells express TCR built of γ and δ chains instead—this subset is commonly called γδ T lymphocytes (Shiromizu and Jancic 2018). In human, one of three δ (δ1, δ2 or δ3) and one of six γ (γ2, γ3, γ4, γ5, γ8 or γ9) chains are used (Shiromizu and Jancic 2018). γδ T cells share characteristics of both adaptive (functional TCR) and innate immunity—they can recognize antigens in an MHC-unrestricted manner and express receptors like natural killer group 2D or Toll-like receptors (Pizzolato et al. 2019; Wu et al. 2014). Human Vδ2 recognizes the so-called phosphoantigens—small phosphorylated molecules like microbial (E)-4-hydroxy-3-methyl-but-2-enyl-pyrophosphate (HMB-PP) (Eberl et al. 2003) or eukaryotic isopentenyl pyrophosphate (IPP) (Tanaka et al. 1995). Vδ1 may recognize a wider range of antigens including some self-antigens like MHC class I polypeptide-related sequence A or UL16-binding protein, that are frequently up-regulated in cancer cells (Kabelitz et al. 2007). γδ T cells are capable of rapid response to a number of threats, including some viral and bacterial infections (Pizzolato et al. 2019). Moreover, they are probably involved in human autoimmune diseases like multiple sclerosis (Zarobkiewicz et al. 2019a), where they may be an important source of early interleukin (IL)-17 that drives further production of IL-17 by Th17 cells (Zarobkiewicz et al. 2019b). Similar to conventional T lymphocytes, γδ T cells can be functionally divided into subsets, e.g., Th1-, Th2-, Th9-, Th17-, Tfh, regulatory T cell (Treg)- and antigen-presenting cell (APC)-like γδ T cells (Pang et al. 2012). The diversity of human γδ T cells is briefly presented in Fig. 1. The majority of γδ T cells express either CD8 or no-TCR-co-receptor (double negative, CD4^–^/CD8^–^)—rarely, they may express CD4 or be double positive (CD4^+^/CD8^+^) (Kadivar et al. 2016).

Asthma is a heterogeneous and serious chronic inflammatory disease of the respiratory system. It is one of the most common airway disorders that affect people in all ages but usually begins in childhood (Frati et al. 2018; Papadopoulos et al. 2019; Wei et al. 2020). Both congenital and acquired factors contribute towards risk of asthma. There is considerable evidence that inflammation is crucial to the pathogenesis of bronchial asthma. Studies attempting to quantify the magnitude of the airway inflammatory response have reported increased eosinophils, basophils, mast-cells, and T lymphocytes in bronchoalveolar lavage fluid (BALF) and blood samples. Among those cells, the Th2 lymphocytes seem to play the fundamental role in asthma pathogenesis. Th2 lymphocytes take part in recruiting eosinophils—by secreting IL-5—and promoting local and systemic synthesis of IgE by producing IL-4. There are a lot of data about the role of αβ T cells in the pathogenesis of asthma; still, mice deficient in αβ T cells were found to make immunoglobulins of all isotypes with high levels of IgE and IgG1, suggesting importance of γδ T cells in asthma development (Lee et al. 2001).

Our current understanding of asthma pathogenesis stems from two major sources—human studies and animal models of asthma. For the latter, mice are most commonly used, especially BALB/c and C57BL/6 strains (Aun et al. 2017). Utilization of animal models makes it possible to use different experimental approaches, e.g., gene-knockouts that otherwise would not be possible. Still, results thereof should be viewed with caution due to important differences between animal and human physiology and immunology (Aun et al. 2017). What should be clearly noted are some major differences between human and mice γδ T cells. First of all, there is a significant difference in *VDJ* segments of TCR used in both species—the Vδ segments distinguish different subsets of human γδ T cells; while in mice, it is the role of Vγ segments. Moreover, subsets described by the similar segment of TCR do not correspond one to the other between species—in short, e.g., the Vγ4 in mice may be functionally significantly different from Vγ4 in humans (Holderness et al. 2013). The majority of human peripheral blood γδ T cells (Vδ2Vγ9) reacts to phosphoantigens; on the other hand, no reactivity to phosphoantigens was so far discovered in mice and rats (Herrmann et al. 2020). Thus, the results of animal studies are not always applicable to humans.

### Blood γδ T Cell Percentage is Lower in Asthmatic Patients

The initial percentage of γδ T cells at 6 months of age seems not to correlate with the risk of developing asthma at the age of 7 (Larsen et al. 2014). On the other hand, a significant decrease in total γδ T cells in peripheral blood was observed in atopic children and atopic adults aged up to 30 (Schauer et al. 1991). Similarly, a significant decrease in peripheral blood γδ T percentage was observed in older (> 65 years old) asthmatic subjects with both mild and severe asthma (Mota-Pinto et al. 2011). This may suggest a role of γδ T cells in the early phases of atopic disease development during childhood. Moreover, a significant decrease in CD8^+^ γδ T lymphocytes was noted in peripheral blood of all atopic patients but the youngest group (< 10 years old) (Schauer et al. 1991).

No difference in the percentage of the total γδ T lymphocytes was observed between asthmatic patients and healthy controls in neither peripheral blood (Bai et al. 2001; Urboniene et al. 2013; Walker et al. 1991) nor induced sputum (Urboniene et al. 2013) or BALF (Krug et al. 2001; Urboniene et al. 2013; Walker et al. 1991). Contrary, in a study by Chen et al. (1996), a significant decrease in the percentage and number of total γδ T cells in peripheral blood of allergic and, to even higher extent, of asthmatic patients was observed, Belkadi et al. (2019) observed similar pattern—a significant decrease in peripheral blood γδ T cell percentage among *Blomia tropicalis* atopic patients. Similarly, in a group of elderly asthmatic patients, a significant decrease in peripheral blood γδ T cells was noted (Todo-Bom et al. 2007). Moreover, Spinozzi et al. (1995) observed significant increase in BALF γδ T cells, both CD4^+^ and double negative, in asthmatic patients, likewise Bai et al. (2001) observed a significant increase in BALF γδ T cells. In fact, most of the BALF CD4^+^ cells in asthmatic patients seem to be γδ T lymphocytes (Spinozzi et al. 1996).

**Fig. 2:**
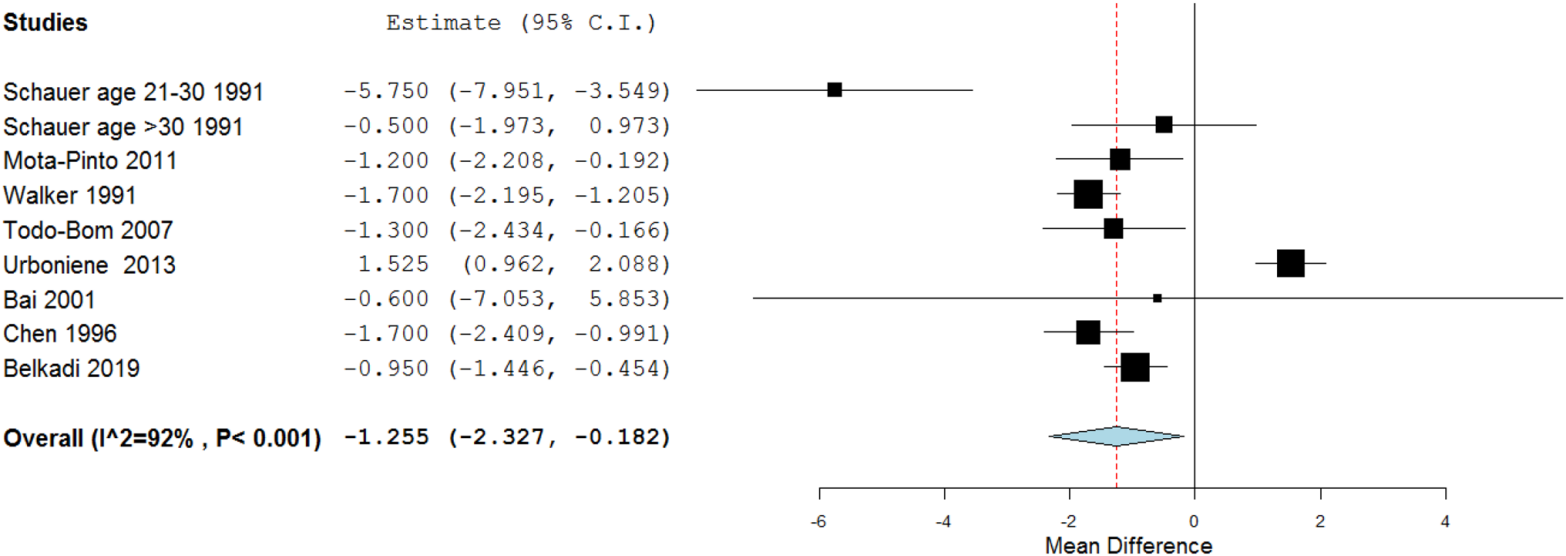
The forest plot of meta-analysis of the percentage of γδ T cells in peripheral blood of asthma patients. A significant decrease thereof can be noted (*p* = 0.022)

Next, we have performed a meta-analysis to better assess the difference in γδ T in peripheral blood, BALF and induced sputum between asthmatic patients and healthy donors. OpenMetaAanalyst was used for calculations (Wallace et al. 2012). If the original article presented data as median, IQR, then an estimation of mean and SD values was performed as proposed by Hozo et al. (2005). Hedges–Olkin method with confidence level 95.0 was used for the analyses (Hedges and Olkin 1985). No conclusive results were obtained for BALF and induced sputum γδ T percentage or absolute numbers. On the other hand, a significant decrease of γδ T percentage in peripheral blood of adult asthma patients was noted (*p* = 0.022; Fig. 2).

Moreover, an up-regulation of Vδ1^+^ γδ T cells and consequent decrease in Vδ2^+^ γδ T cells in BALF of asthmatic patients was noted (Bai et al. 2001). Some signs of monoclonal–oligoclonal type of expansion were noted in BALF γδ T cells of asthmatics as well (Bai et al. 2001). Total γδ T and CD8^+^ γδ T cells were found to be significantly increased in induced sputum of asthmatic patients during exacerbation (Hamzaoui et al. 2002). This rise can be mostly ascribed to the increase in Vδ1 subset as the Vδ2 resembles that of the control group (Hamzaoui et al. 2002). Moreover, the activation markers (CD25) were expressed more frequently—on up to one sixth of γδ T cells (Hamzaoui et al. 2002). γδ T cells from induced sputum of asthmatic patients during exacerbation naturally exhibit higher FasL expression (on approximately one sixth of them) than the control samples and are significantly more cytotoxic (Hamzaoui et al. 2002).

Concluding, it seems that adult asthmatic patients tend to have lower percentages of γδ T cells in peripheral blood—this should, however, be tested on some larger groups as the current data are still inconclusive. Apart from total γδ T percentage, the internal balance between various γδ T cell subsets, e.g., Vδ1/Vδ2 balance seems to be of importance in asthma. Therefore, further studies should focus also on an in-depth description of functional landscape within the γδ T compartment.

### γδ T Depletion/Knockout Lowers BALF Eosinophilia and Serum IgE Levels in Murine Model of Asthma

Significantly lower number of total leukocytes, eosinophils and lymphocytes in BALF was observed in γδ T-deficient mice than in wild-type ones (Schramm et al. 2000; Svensson et al. 2003); moreover, the percentage of eosinophils and lymphocytes dropped, while that of macrophages nearly doubled (Schramm et al. 2000). This effect is less pronounced when anti-TCRγδ antibodies are used to deplete γδ T cells than when the knockout mice are used (Schramm et al. 2000). A significant decrease in BALF B cells was noted in ovalbumin (OVA)-challenged γδ-knockout mice (Svensson et al. 2003). In addition, a significant drop in BALF level of OVA-specific IgA and IgG was also noted, suggesting attenuated immunoglobulin synthesis in airways (Svensson et al. 2003). This may suggest either subtotal depletion in the former case or that the function of γδ T lymphocytes is important for the proper maturation of αβ T cells. In a study by Tamura-Yamashita et al. (2008), the number of total leukocytes in BALF in γδ-knockout mice remained similar to that of wild-type ones, but the percentage of eosinophils significantly dropped and the number of macrophages rose. Similarly, a decrease in eosinophilia and IgE level was noted in γδ-knockout mice in a model of *B. tropicalis* asthma (Belkadi et al. 2019). Finally, the percentage of γδ T cells in BALF of asthmatic mice rises significantly post OVA challenge, but nevertheless remains low (Landgraf and Jancar 2008).

This suggests a significant role of γδ T cells in regulation of IgE production and influx of eosinophils to airways.

### γδ T Cells Take Part in Regulation of IgE Production

Indeed, the influence of γδ T cells on IgE production has been briefly researched in murine models of asthma. The CD8^+^ γδ T cells seem to be capable of significant suppression of IgE secretion in mice after repeated exposure to OVA aerosol; this is probably mediated by interferon (IFN)-γ (Huang et al. 2009, 2013; McMenamin et al. 1994). Those cells tend to express Vγ4 (Huang et al. 2009, 2013) and most of them Vδ5 chains (Huang et al. 2009). Similar results were obtained for Brown Norway rats (McMenamin et al. 1995). The protective influence of some γδ T subsets in mice was further evaluated in knockout models. Vγ4 and Vγ6-knockout mice exhibited high levels of serum IgE without any treatment similarly to wild-type mice after OVA sensitization (Huang et al. 2009). Moreover, treatment with anti-Vγ4 antibody significantly increased total serum IgE level in wild-type mice after OVA sensitization (Huang et al. 2009). On the other hand, the Vγ1^+^ γδ T cells seem to increase the total IgE level as well as the OVA-specific IgE after OVA sensitization in mice (Huang et al. 2009). Nevertheless, total γδ T lymphocytes seem to generally promote IgE suppression (Huang et al. 2009).

On the other hand, according to Seymour et al. (1998), γδ T lymphocytes and IFN-γ are not required for IgE suppression. This is contrary to the previous results of McMenamin et al. (1994, 1995), who proved that adoptive transfer of γδ T cells even in low number caused significant decrease of OVA-induced IgE production and by Huang et al. (2009), who observed that in IFN-γ-knockout mice such effects were not observed. It seems that there is no need for the direct contact of γδ T cells with the antigen—they can be induced by activated splenocytes, mostly non-T CD11c^+^ (Huang et al. 2013). During this process, unprocessed or partially processed allergen can be transferred to a small part of γδ T cells (Huang et al. 2013). This subpopulation is mostly MHC II^+^ (Huang et al. 2013) and, thus, should be labeled as APC-like γδ T (Pang et al. 2012). Moreover, the APC-like γδ T cells seem to be critical for the γδ T-mediated IgE-production suppression (Huang et al. 2013). The γδ T-deficient mice are capable of IgE production following proper OVA immunization in a similar manner to the wild-type mice (Korsgren et al. 1999; Tamura-Yamashita et al. 2008; Wang and HayGlass 2000; Zuany-Amorim et al. 1998), but the total IgE (Schramm et al. 2000) and OVA-specific IgE (Svensson et al. 2003; Tamura-Yamashita et al. 2008) may be significantly lower in γδ T-deficient mice. The γδ T-mediated IgE suppression seems to be allergen specific (Huang et al. 2013). This suggests that γδ T cells are important for the successful immunotherapy, but are not necessary for the allergic reaction to occur.

### Airway Hyperresponsiveness is Partially Governed by the Vγ1 and Vγ4 Balance in Mice

**Fig. 3:**
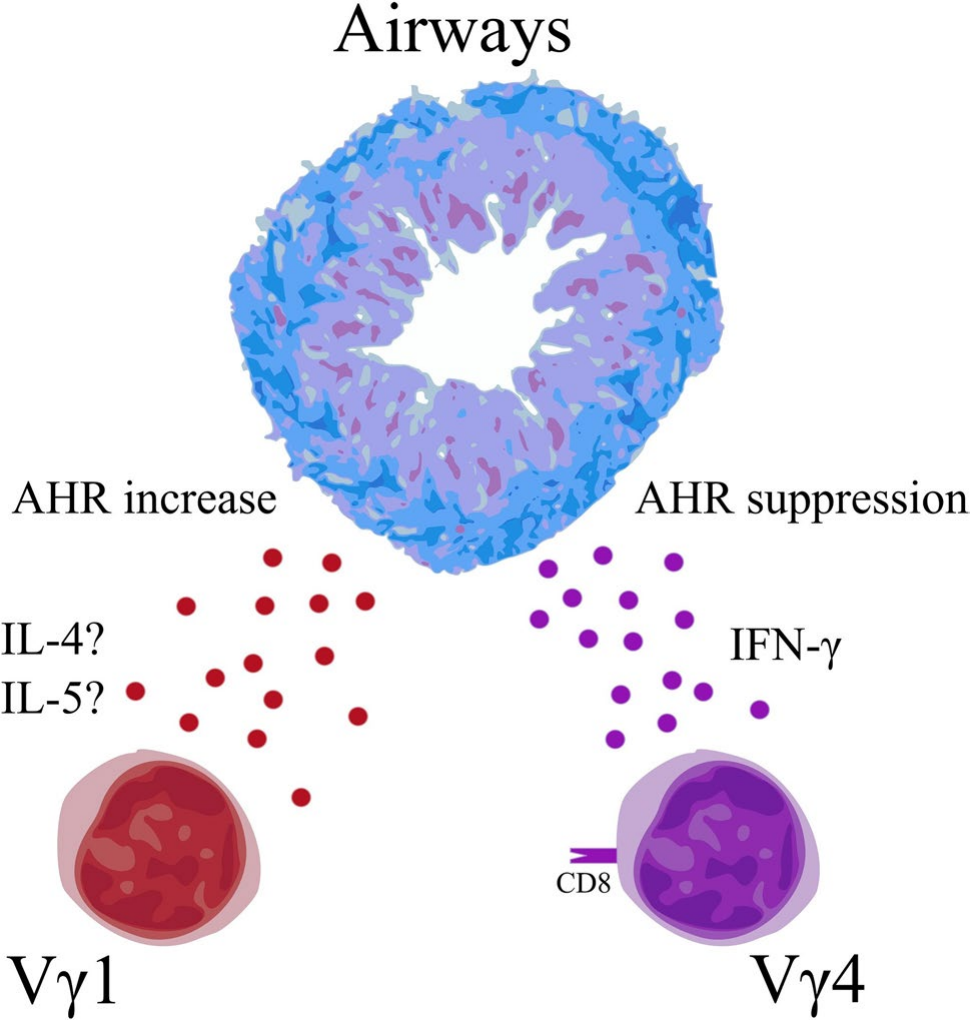
The γδ T subsets and airway hyperresponsiveness in the mouse model of asthma. Vγ1, probably by secreting Th-2-type cytokines, promotes airway hyperresponsiveness (AHR), while Vγ4 seems to decrease AHR by secreting IFN-γ. Thus, the former aggravates symptoms of disease in murine model of asthma, while the latter alleviates them (Belkadi et al. 2019; Cook et al. 2008; Cui et al. 2003; Hahn et al. 2004; Jin et al. 2009; Lahn et al. 2002)

Airway hyperresponsiveness (AHR) is a predisposition of airways to contract in response to a stimulus that does not produce such an effect in a healthy subject (Chapman and Irvin 2015). Despite being introduced nearly half-century ago, AHR is still one of the core concepts in the current understanding of asthma pathogenesis. In mouse model of OVA-induced asthma, depletion of γδ T cells after sensitization leads to an increase in AHR (Lahn et al. 1999; Schramm et al. 2000), while depletion of αβ T cells leads to total lack of response to OVA challenge (Schramm et al. 2000). On the other hand, total depletion of γδ T cells before OVA sensitization leads to a significant decrease of AHR; similar effect was observed when anti-Vγ1 antibody was used, but nothing changed after Vγ4-depletion (Hahn et al. 2004). Similarly, a significantly decreased AHR was observed in γδ-knockout mice after *B. tropicalis* challenge, and adoptive transfer of wild-type mouse Vγ1 γδ T cells completely reverses this process; this was not observed in the case of IL-4-knockout mouse Vγ1 γδ T cells (Belkadi et al. 2019). This suggests that γδ T may play an important, but not crucial role in establishing asthma-related AHR, the Vγ1^+^ cells seem especially important therein, while the Vγ4 seem not to be involved. The difference in effect between those two times of depletion may indicate that the AHR-aggravating Vγ1 cells are mostly needed at the time of challenge, probably for their IL-4 production, while the AHR-suppressing Vγ4 are required post-challenge to perform their action. This is partially supported by the fact that even though the Vγ4 cells are the major subset of γδ T lymphocytes in normal mice lungs, they also are strongly induced by the OVA sensitization (Hahn et al. 2003). Vγ1 and Vγ4 opposition in mice is presented in Fig. 3.

Similar balance between Vγ1 and Vγ4 has been described in other mice pathologies (Born et al. 2010). While Vγ1 plays a positive and Vγ4 negative role in one disease, the opposite may be observed in yet another case.

### Vγ4-Dependent AHR Decrease is Probably Mediated by IFN-γ

The Vγ1Vδ5 γδ T lymphocytes seem to promote increased AHR in mice model (Jin et al. 2009). Their actions seem to be not related to the typical Th2 cytokines as they rarely produce IL-4 or IL-13 (Jin et al. 2009). Nevertheless, a significant decrease in BALF IL-13 and IL-5 and increase in IL-10 were noted in γδ-deficient mice; after adoptive transfer of Vγ1 γδ T cells, the levels of those cytokines normalized in relation to the wild-type asthmatic mice (Hahn et al. 2004). Functional studies indicate that their potential to increase AHR is at least partially dependent on the external source of TNF-α, IFN-γ and IL-4 (Jin et al. 2009). The AHR-enhancing subset of γδ T cells can be developed by either the influence of those three cytokines or by stimulation with OVA; for both ways, the CD8^+^ dendritic cells are probably necessary (Cook et al. 2008; Jin et al. 2009). Nevertheless, the AHR-enhancing subset probably does not require antigen priming; its action is also probably further promoted by invariant natural killer T cell cells (Jin et al. 2007). The Vγ4^+^ AHR-decreasing γδ T lymphocytes requires the CD8^+^ dendritic cells to perform their function properly (Cook et al. 2008). Contrary to the Vγ1^+^ AHR-enhancing subset, the Vγ4^+^ AHR-decreasing subpopulation does require the allergen-driven induction and activation to perform its suppressive role, but what is important the allergen may be mismatched—ragweed-, BSA- and OVA-induced cells exhibited similar suppressive effect in OVA-sensitized mice (Jin et al. 2005). The use of aerosolized anti-γδ T antibody leads to aggravated AHR and increased airway inflammation, while use of anti-Vγ4 exerts similar effects, but with decrease in inflammation and the effect of aerosolized anti-Vγ1 is yet to be discovered—one study found no effect on AHR, but in another it seems to decrease both the AHR and the airway inflammation (Lahn et al. 2002, 2004). The Vγ4-knockout mice exhibit significantly increased AHR (Lahn et al. 2002). This suggests that the AHR-regulating properties of both the suppressive Vγ4 and the enhancing Vγ1 subsets can be exerted only by the locally present cells. An important part of the Vγ4 subset is CD8^+^ and produces IFN-γ, which seems crucial for the ability of Vγ4 to suppress AHR (Lahn et al. 2002). Long-term challenge with OVA caused nearly complete lack of AHR, the depletion of either total γδ or only Vγ4 subset restored the normal airway response, suggesting an important protective role thereof (Cui et al. 2003).

The γδ-knockout mice after OVA challenge show significantly lower late airway response in contrast to the early airway response that is similar to that observed in wild-type ones (Tamura-Yamashita et al. 2008). On the other hand, the adoptive transfer of IFN-γ^+^ CD8^+^ γδ T cells potently inhibits the late airway response and BALF eosinophilia in rats after OVA challenge (Isogai et al. 2007); this effect seems to be noticeable only when the cell donor was OVA naive (Isogai et al. 2003). Moreover, the percentage of major basic protein-positive eosinophils in BALF is also significantly lower (Isogai et al. 2003, 2007); similarly, the level of IL-4 and IL-5 (Isogai et al. 2003, 2007) and IL-13 (Isogai et al. 2003) mRNA in BALF cells and cysteinyl leukotrienes in BALF also drops significantly (Isogai et al. 2007). An increase in IFN-γ mRNA was also noted, implying a possible shift towards Th1 response (Isogai et al. 2003).

### Viral infections Affect Airway γδ T Cells an2 Change the Course of Asthma Exacerbation

Viral infections of upper respiratory tracts belong to the most common causes of acute asthma exacerbations, both in adults and children (Dougherty and Fahy 2009). Although γδ T cells are already increased in BALF of asthmatic patients, they are even further increased during viral-induced asthma exacerbation with their number correlating with AHR, eosinophil count in BALF and airway obstruction (Glanville et al. 2013). The respiratory syncytial virus (RSV) infection seems to promote FasL-dependent apoptosis of γδ T cells in mice lungs, leading to alleviated symptoms of OVA-induced asthma (Zeng et al. 2014). Moreover, RSV infection, that precedes the OVA induction of asthma, leads to significantly milder course; this could possibly be ascribed to the shift in Th1/Th2 balance among γδ T cells, namely the elevated expression of IFN-γ (Th1-like γδ T) and decreased that of IL-4 (Th2-like γδ T) (Zhang et al. 2013b). This balance is, however, unaffected if the infection occurs post OVA immunization (Zhang et al. 2013b). Finally, pan-γδ T depletion leads to a significant increase in AHR, and both neutrophil and lymphocyte (including rise in Th2 cells) count in BALF during viral-induced exacerbation in OVA model of asthma in mice (Glanville et al. 2013). This suggests the complexity of γδ T involvement in the pathogenesis of asthma. Clearly, depending on the predominant functional landscape within γδ T cells, they can either alleviate or aggravate symptoms.

### Th1-Like γδT are Either Decreased or Increased Depending on the Stimulus

Asthma is traditionally regarded as Th2-driven, though Th1-related response is also believed to be important for the maintenance of chronic inflammation (Ngoc et al. 2005). Moreover, higher than usual Th1 response has been linked to psychological problems like anxiety or depression among asthma patients (Zhu et al. 2016). In fact, lung-infiltrating IFN-γ^+^ γδ T (Th1-like) lymphocytes are significantly expanded in OVA-induced asthma model and viral-mediated exacerbation thereof (Glanville et al. 2013). In contrast to Th2-like γδT, Th17-like γδT and Treg-like γδT, this effect slightly diminishes over time (Glanville et al. 2013). Still, in another study, a significant decrease in IFN-γ^+^ γδ T cells in lungs of OVA-induced asthmatic mice was observed; this effect was significantly attenuated by the inhalation of inactivated *Mycobacterium phlei* (Zhang et al. 2013a). Moreover, lung-infiltrating γδ T cells are rarely IFN-γ^+^ in the murine model of asthma (Murdoch and Lloyd 2010).

The IFN-γ^+^ γδ T cell percentage is significantly decreased in peripheral blood of asthmatic patients (Zhao et al. 2011). Similarly, a significant decrease in BALF IFN-γ^+^ γδ T and IL-2^+^ γδ T cells was observed in asthmatic subjects post allergen challenge, no such change was noted among healthy subjects, though the initial values in both groups were similar (Krug et al. 2001). Nevertheless, in a recent study on the *B. tropicalis* allergic patients—a significant up-regulation of IFN-γ^+^ γδ T cells in peripheral blood was observed (Belkadi et al. 2019).

Studies on both animal model and human asthmatic subjects show the heterogeneity of responses—the prevalence of Th2-like γδ T differs between various settings, possibly being partly dependent on the exact nature of stimulus.

### Airway Epithelial γδ T Cells are Th2-Skewed in Asthmatic Patients

Despite being a minor population in peripheral blood and lymph nodes, γδ T are one of the major lymphocyte subsets at epithelial barriers, including airway epithelium (Born et al. 2000). γδ T lymphocytes comprise about one fourth of total epithelial infiltrating lymphocytes in nasal cavities of allergic patients (Pawankar et al. 1996). Half of them is double negative, one fourth CD4^+^ and the remaining fourth is CD8^+^ (Pawankar et al. 1996). Among those γδ T lymphocytes, the Vγ1 (approx. two thirds) and Vδ1 (approx. four fifths) prevail (Pawankar et al. 1996). Only marginal part of nasal epithelial γδ T cells produces IFN-γ (Th1-like γδ T); while one third secretes IL-4 and one fourth IL-5, which suggest a significant skew towards Th2-like γδ T cells in allergic patients (Pawankar et al. 1996). No significant difference in the total number of γδ T cells in bronchial mucosa and submucosa was observed in asthmatic patients (Fajac et al. 1997). No co-localization with heat shock protein (HSP)-60-, HSP-70- or HSP-80-positive epithelial cells was noted either (Fajac et al. 1997).

On the other hand, γδ T cells seem to be important for the bronchial infiltration in OVA-induced animal asthma model—the γδ T-deficient mice tend to have lower Th, Tc (Korsgren et al. 1999; Zuany-Amorim et al. 1998) and eosinophil bronchial infiltration (Korsgren et al. 1999; Lahn et al. 1999; Zuany-Amorim et al. 1998) as well as attenuated total inflammatory infiltration (Schramm et al. 2000). Moreover, treatment with anti-TCRγδ antibody during the resolution of allergic response leads to prolonged eosinophilic and Th2 airway infiltration in an OVA-induced murine asthma model; similarly, it prevents the drop in IL-4 content, typically observed during resolution phase (Murdoch and Lloyd 2010). This phenomenon may probably be related to the Vγ1 and Vγ4 balance—while the former seems to be important for the onset of symptoms and airway infiltration, the latter may have a significant role in symptom resolution.

Altogether it once again suggests the complexity of the γδ T compartment in asthma. Current data on airway γδ T cells in asthmatic patients show a significant skew towards Th2 type of response, thus suggesting a possible importance thereof in asthma pathogenesis (Tables 1, 2).

**Table 1:** Involvement of γδ T cells in murine asthma

No.	Model	Short summary	References
1	Fungal asthma: *Aspergillus fumigates*; C57BL/6 mice	γδ T cells among others as source of IL-22;	Reeder et al. (2018)
2	Ozone exposure; *db/db* obese mice and C57BL/6 mice	Ozone-related release of IL-33 promotes Th2-type of response in γδ T cells	Matthews et al. (2017)
3	OVA-induced asthma; BALB/c mice. Treatment with *Mycobacterium phlei*	*M. phlei* regulates IL-17 production by γδ T cells by lowering the expression of IL-23R	Ming et al. (2017)
4	House dust mite-induced asthma; various mice strains incl. C57BL/6, Atg5-deficient mice	Disruption of autophagy promotes IL-17A production by γδ T cells	Suzuki et al. (2016)
5	OVA-induced asthma; BALB/c mice, RSV infection pre- or post-induction	pre-induction RSV infection partially suppresses asthma due to FasL-dependent apoptosis of pulmonary γδ T cells	Zeng et al. (2014)
6	House dust mite or cockroach-induced asthma; C57BL-6 mice + transgenic mice	Both stimuli induced inflammatory response. Cockroach challenge induced IL-6 trans-signaling and thus IL-17 production in γδ T cells	Ullah et al. (2015)
7	OVA-induced asthma—with no adjuvant or aluminum or complete Freud adjuvant; BALB/c mice	Stimulation with OVA + complete Freud adjuvant promotes Th17-like γδ T cells. Th17-like γδ T cells alleviate symptoms and significantly lower AHR	Nakada et al. (2014)
8	Rhinovirus-mediated exacerbation of OVA-induced asthma, BALB/c mice; anti-TCRγδ treatment	Lung γδ T during infection produce IL-17 and IFN-γ; they alleviate asthma-symptoms	Glanville et al. (2013)
9	OVA-induced asthma; BALB/c mice; RSV infection	Prior RSV infection decreases the number of lung-infiltrating γδ T and promotes Th1 over Th2-response in γδ T cells	Zhang et al. (2013b)
10	OVA-induced asthma; BALB/c mice; *M. phlei* treatment post-induction	Attenuated airway inflammation. Increase in Treg-like and Th1-like γδ T cells	Zhang et al. (2013a)
11	cytokine-induced asthma-like symptoms; BALB/c, C57BL/6 mice and knockout mice	Th17-like γδ T cells seem to alleviate symptoms of asthma	Kinyanjui et al. (2013)
12	OVA-induced asthma; C57BL/6 mice and various knockout mice; transfer of γδ T cells	Vγ4^+^ γδ T cells in mice are capable of antigen-specific regulation of IgE production	Huang et al. (2013)
13	OVA-induced asthma; BALB/c and C57BL/6, and IL-17-knockout C57/BL/6	Th17-like γδ T cells are significantly expanded in lungs and are important for symptom resolution	Murdoch et al. (2010)
14	OVA-induced asthma, C57BL/6 mice and knockout mice; γδ T knockout, γδ T depletion, adoptive transfer	Vγ4^+^ Vδ5^+^ CD8^+^ γδ T cells suppress, while Vγ1^+^ γδ T cells enhance IgE production	Huang et al. (2009)
15	OVA-induced asthma; C57BL/6 and several knockout mice; knockout and adoptive transfer	Vγ1^+^ γδ T cells are important enhancers of AHR; they probably have some regulatory capability over Th cells, promoting Th2 phenotype	Jin et al. (2009
16	OVA-induced asthma; BALB/c mice and γδ T-knockout mice	γδ T cells are important for late AHR and airway inflammation	Tamura-Yamashita et al. (2008)
17	OVA-induced asthma; C57BL/6 and several knockout mice; Vγ4 antibody-mediated depletion, adoptive transfer	CD8^+^ dendritic cells are required for the development of AHR-promoting Vγ1 γδ T cells	Cook et al. (2008)
18	OVA-induced asthma; Brown Norway rats; adoptive transfer	IFN-γ expressing CD8^+^ γδ T cells are important for inhibition of late AHR	Isogai et al. (2007)
19	OVA-induced asthma, C57BL/6 mice and knockout mice; adoptive transfer; iNKT antibody-mediated depletion	Vγ1 cells require interactions with iNKT cells to be able to promote AHR	Jin et al. (2007)
20	OVA-induced asthma; C57BL/6 mice and several knockout mice; adoptive transfer; Vγ4 antibody-mediated depletion	Vγ4 subset is only capable of suppression of AHR when it was previously activated with antigen, the latter can be different from AHR-causing allergen	Jin et al. (2005)
21	OVA-induced asthma; C57BL/6 mice and several knockout mice; antibody-mediated depletion of pan γδ T or Vγ1 or Vγ4 γδ T cells; adoptive transfer	Vγ4 γδ T cells suppress AHR while Vγ1 γδ T cells promote AHR	Hahn et al. (2004)
22	OVA-induced asthma, C57BL/6 mice and knockout mice; adoptive transfer; antibody-mediated depletion of Vγ4 γδ T cells	Vγ4 γδ T cells are capable of suppressing AHR	Hahn et al. (2003)
23	OVA-induced asthma, C57BL/6 mice and knockout mice;	γδ T-deficient mice have significantly lower OVA-specific IgE and eosinophilia	Svensson et al. (2003)
24	OVA-induced asthma, C57BL/6 mice and knockout mice; antibody-mediated depletion of various T cells	Lung Vγ4 γδ T suppress AHR, for that they require IFN-γ and MHC class I	Lahn et al. (2002)

Results of major studies on animal models of asthma published during the last 20 years

iNKT: invariant natural killer T cell

**Table 2:** Results of major studies on γδ T involvement in human asthma

No.	Participants	Study design	Major results	References
1	Adults: 60 asthmatic and 24 healthy subjects	Induced sputum, bronchial bioptates, BALF and peripheral blood T cells were analyzed with flow cytometry	No difference in Th17-like γδ T cells between asthmatic and healthy subjects	Hinks et al. (2015)
2	Adults; 9 asthmatic, 15 healthy	Participants were intranasally infected with rhinovirus; enumeration of γδ T in BALF and peripheral blood	BALF γδ T are significantly more numerous in asthmatic patients, 4 days post infection they increase even further	Glanville et al. (2013
3	Adults; 20 patients with chronic obstructive pulmonary disease, 18 with asthma and 14 healthy	Enumeration of T cells (incl. γδ T) in induced sputum, peripheral blood and BALF	No significant differences between asthmatic and healthy subjects were noted in γδ T percentage or absolute number	Urboniene et al. (2013)
4	Adults > 65 years; 95 asthmatic and 58 healthy subjects	Flow cytometric analysis of T cell subsets in peripheral blood	γδ T cell percentage was significantly decreased in asthmatic patients	Mota-Pinto et al. (2011)
5	Adults; 29 asthmatic, 12 healthy subjects	Flow cytometric analysis of IFN-γ, IL-4 and IL-17 expression in T cells	Increase in IL-4 expression and decrease in IFN-γ in γδ T cells	Zhao et al. (2011)
6	Adults; 64 asthmatic (each having asthma > 30 years), 61 healthy subjects (41 older adults; 20 younger adults)	Flow cytometric analysis of T cell subsets in peripheral blood	γδ T cells are lowered in asthmatic patients compared to both age-matched and younger control group	Todo-Bom et al. (2007)
7	Adults; 10 asthmatic and 10 healthy subjects	Flow cytometric analysis of T cell subsets in induced sputum; cytokine expression; γδ T-mediated cytotoxicity	Significantly higher percentage of γδ T cells in induced sputum among asthmatic patients; significant increase in TNF-α, IL-4 and IL-10 in γδ T cells form asthmatic patients	Hamzaoui et al. (2002)
8	Adults: 11 with mild asthma and 9 healthy subjects	Flow cytometric analysis of cytokine expression by T cells from BALF. Segmental allergen challenge	Increased IL-5 and IL-13 expression in γδ T without prior segmental challenge	Krug et al. (2001)
9	Adults: 7 asthmatic and 7 healthy controls	Flow cytometric analysis of BALF and peripheral blood γδ T cell percentage. Vδ clonality was assessed with RT-qPCR	Significantly higher percentage of γδ T cells in BALF, higher expression of Vδ1 gene with limited oligoclonality in BALF of asthmatic patients	Bai et al. (2001)
10	Adults: 10 asthmatic and 9 healthy individuals	Immunohistochemistry—analysis of T cells in bronchial bioptates	No significant difference in bronchial γδ T cells between groups	Fajac et al. (1997)
11	Adults: 17 asthmatic, 22 allergic non-asthmatic, 23 healthy subjects	Flow cytometric analysis of T cell subsets in peripheral blood	Significantly lower γδ T percentage in peripheral blood of asthmatic patients	Chen et al. (1996)
12	6 children and 4 adult asthmatic patients; 10 age-matched healthy subjects; 5 sarcoidosis patients	Flow cytometric analysis of γδ T cell subsets in BALF; in vitro cytokine production	Significantly higher absolute γδ T count in BALF of asthmatic subjects. Significantly higher IL-4 expression in asthmatic patient-derived γδ T cells	Spinozzi et al. (1995)
13	20 children with atopic asthma; 11 children with atopic dermatitis, 18 adults with atopic dermatitis; controls: 21 children and 17 adults	Flow cytometric analysis of γδ T cells in peripheral blood	Significantly lower γδ T percentage among young atopic patients; concurrently, significantly higher expression of CD8 on γδ T cells in atopic patients	Schauer et al. (1991)
14	Adults: 17 asthmatic and 10 healthy	Flow cytometric analysis of T cell subsets in peripheral blood and BALF	No difference in BALF γδ T cell percentage and absolute count	Walker et al. (1991)

### Th2-Like γδ T Constitute Only a Minor Population in Animal Model, but are Significantly Increased in Asthmatic Patients

Th2 type of response is one of the main concepts in asthma pathogenesis; here, we consider all γδ T cells producing any of Th2 cytokines (IL-4, IL-5 or IL-13) as Th2-like γδ T (Barnes 2001). IL-4^+^ γδ T cells, although being expanded in OVA-induced asthma and viral-mediated exacerbation, constitute only a minor subset of total γδ T lymphocytes in mice lungs (Glanville et al. 2013). Generally, γδ T cells secreting either of Th2-related cytokines are scarcely present in lungs of asthmatic mice (Murdoch and Lloyd 2010). Moreover, only a slight decrease in BALF IL-4 content was noted in γδ T-deficient mice following OVA challenge (Zuany-Amorim et al. 1998). Nevertheless, in vivo administration of IL-4 to γδ T-deficient mice restored the wild type-like effect of OVA challenge, thus suggesting an importance of this small subset (Zuany-Amorim et al. 1998). In fact, OVA seems to stimulate IL-4 expression in mouse γδ splenocytes (Tamura-Yamashita et al. 2008). BALF concentration of IL-5 is significantly lower in γδ T-deficient mice after OVA immunization compared to wild type—suggesting the importance of γδ T for overall IL-5 production (Zuany-Amorim et al. 1998). On the other hand, stimulation of bronchial biopsy cultures with anti-TCRγδ leads to only marginal IL-5 production when compared to allergen, αβ TCR stimulation or pan-T stimulation with anti-CD3 (Jaffar et al. 1999). This suggests some importance of γδ T cells for both IL-4 and IL-5 production, but they may rather play a regulatory role as they rarely produce significant amounts of those cytokines themselves. Nevertheless, in a mouse model of *B. tropicalis* house-dust-mite asthma, a significant up-regulation of IL-4 and IL-13 production was noted among lung-infiltrating Vγ1 γδ T cells (Belkadi et al. 2019). Similarly, a higher percentage of IL-4-producing γδ T cells in peripheral blood was noted among *B. tropicalis* allergic patients, though it was still very low (Belkadi et al. 2019).

Air pollution may trigger asthma exacerbation—both the particulate matter (mostly PM2.5) and ozone; the latter is believed to be the cause of up to 20% of all asthma-related emergency room visits worldwide (Anenberg et al. 2018). Obesity is yet another risk factor—both for asthma in general and for severe asthma (Peters et al. 2018). Ozone exposure leads to an increase in total γδ and IL-13^+^ γδ cells within lungs of obese mice, the latter seems of particular importance in the pathogenesis of ozone-related asthma exacerbation in obese subjects (Mathews et al. 2017). The ozone-related increase in BALF IL-13 and IL-5 levels was significantly lower in TCRδ-deficient obese mice which further confirms the importance of γδ T lymphocytes as the source of IL-13 in exacerbation (Mathews et al. 2017). Generally, ozone exposure triggers release of IL-33 in airways; this cytokine is responsible for ozone-related AHR (Kasahara et al. 2019). The up-regulation of IL-13 and IL-5 are most probably related to IL-33; its receptor (ST2) is present on γδ T lymphocytes (Mathews et al. 2017). Those effects were not observed in non-obese mice.

Pro- and anti-apoptotic balance is one of the main mechanisms in T cell biology—significant change towards any of those directions may lead to either autoimmunity or cancer and immunodeficiency (Murali and Mehrotra 2011). Indeed, impairment of T cell apoptosis was suggested as one of the asthma pathomechanisms (Spinozzi et al. 2008). Allergen-specific Th2-like CD30^+^ γδ T lymphocytes are expanded in the BALF of asthmatic patients (Spinozzi et al. 1995). CD30 promotes expression of anti-apoptotic Bcl-2 family proteins, namely Bcl-2 and Bcl-xl; increasing survival and proliferation by impairing apoptosis (Banjara et al. 2020; Wang et al. 2020). Similarly, IL-4^+^ γδ T lymphocytes are significantly increased in peripheral blood of asthmatic patients, compared to healthy control (Zhao et al. 2011) and in induced sputum (during asthma exacerbation) after short-term phytohemagglutinin stimulation (Hamzaoui et al. 2002). A significantly higher percentage of IL-5^+^ γδ T cells and IL-13^+^ γδ T cells was noted in BALF of asthmatic patients versus healthy controls, no difference was observed in IL-4^+^ γδ T cells (Krug et al. 2001). The values remained steady following the allergen challenge in both healthy and asthmatic subjects (Krug et al. 2001).

### Lung-Infiltrating γδ T Predominantly Express IL-17 in Murine Model of Asthma

In healthy mouse lungs, γδ T lymphocytes are relatively abundant, consisting up to 20% of total lymphocytes in that niche (Born et al. 2000). Still, their number is increased in lungs of mice with experimentally induced asthma, both in the case of house-dust-mite and cockroach challenge (Belkadi et al. 2019; Ullah et al. 2015). Moreover, the γδ T lymphocytes are also increased in the lungs in both the mice model of OVA-induced asthma (Cui et al. 2003; Glanville et al. 2013; Zhang et al. 2013b) and RSV-related exacerbation of asthma (Glanville et al. 2013; Zhang et al. 2013b). The number of lung-infiltrating γδ T cells is the higher the longer airway challenge to OVA lasts (Cui et al. 2003). However, no significant influx of γδ T cells was observed after OVA challenge, when the RSV infection preceded it (Zhang et al. 2013b). In animal model of house-dust-mite asthma, lung-infiltrating γδ T cells express IL-6R (Ullah et al. 2015) which predisposes them to either Th17- or Th2-like roles (Su et al. 2017). Similarly, in a mice model of OVA-induced asthma, the majority of lung-infiltrating γδ T cells express IL-17, while there are scarcely any expressing either IFN-γ or IL-4 (Murdoch and Lloyd 2010). A significant up-regulation (fivefold) of lung-infiltrating γδ T cells was observed in Bim-deficient^1^ mice—no significant change was observed after OVA sensitization—this implies the importance of apoptosis for the regulation of lung-infiltrating γδ T lymphocytes as the up-regulation was strikingly stronger in the case of γδ T cells than αβ CD4^+^ or CD8^+^ (Pierce et al. 2006). Studies on murine model of asthma suggest that in the course of disease, there may be a significant increase in pulmonary γδ T cells, mostly those producing IL-17. This may possibly be attributed to some disregulations in γδ T apoptosis.

### Th17-Like γδ T May Reduce AHR in Murine Model of Asthma

According to recent studies, IL-17 is an important cytokine for the pathogenesis of asthma and its exacerbation in at least some asthmatic patients, especially those with severe asthma (Chakir et al. 2003; Iwanaga and Kolls 2019), the effect of IL-17 on asthma seems to be dose dependent with low doses increasing and high doses decreasing the AHR (Kinyanjui et al. 2013). IL-23 is one of the major regulators of Th17 type of response—it promotes expansion and survival of Th17 cells, mostly by activation of STAT4 (Khan and Ansar Ahmed 2015). Indeed, an increase of IL-23 levels in lungs of mice after OVA challenge during the acute phase of allergic response was observed (Murdoch and Lloyd 2010). Moreover, an increase in IL-23R^+^ γδ T cells was observed in BALF in murine model of asthma (Ming et al. 2017). This may be related to a further increase in IL-17^+^ cells and concomitant symptom resolution.

An increase in the percentage of Th17-like γδT was observed in BALF in murine model of asthma (Belkadi et al. 2019; Ming et al. 2017; Murdoch and Lloyd 2010; Zhang et al. 2019). Th17-like γδ T cells are even more numerous than Th17 lymphocytes in lungs during OVA-induced asthma in mice (Murdoch and Lloyd 2010). Th17-like γδT were found to be the major source of IL-17A in lungs of mice after cockroach challenge (Ullah et al. 2015). IL-17A production seems to be stimulated by IL-6 trans-signaling—IL-6 first binds to the soluble IL-6R (sIL-6R) and then that complex associates with glycoprotein 130 to transduce the signal (Rose-John and Heinrich 1994; Ullah et al. 2015). This effect was, however, not observed in house-dust-mite asthma model (Ullah et al. 2015), which may be related to the fact that IL-6 trans-signaling is relevant in only a group of asthmatic patients, recently marked as a distinct subset (Jevnikar et al. 2019). Moreover, IL-13 may also stimulate γδ T cells to produce IL-17 (Kinyanjui et al. 2013).

Activation of Th17-like γδT cells in mice leads to reduced AHR (Kinyanjui et al. 2013; Nakada et al. 2014), decreased eosinophil, but increased neutrophil airway infiltration (Nakada et al. 2014). Similarly adoptive transfer of γδ T cells, among which no less than 75% are IL-17^+^, or of only the Vγ4^+^ Th17-like γδT cells leads to symptom attenuation and significant decrease in Th2-related cytokines (IL-4, IL-13) and decreased lung infiltration, and increased neutrophil airway infiltration. Finally, among macrophages, the alveolar ones were increased and tissue ones decreased (Murdoch and Lloyd 2010). This effect seems to be IL-17-driven as treatment with IL-17 instead of cells leads to similar effects (Murdoch and Lloyd 2010). Both treatments cause also a significant decrease in eotaxin-1 and CCD20 and significant increase in CCL2 in lungs (Murdoch and Lloyd 2010). The adoptive transfer of OVA-sensitized Th17 cells does not cause similar changes as only reduced eosinophilic infiltration was observed thereafter (Murdoch and Lloyd 2010). Autophagy is somehow linked to asthma pathogenesis—increased autophagy was noted in bronchial tissue from asthmatic patients, autophagy is also important for IL-13-dependent up-regulation of mucus production (Dickinson et al. 2016; Jyothula and Eissa 2013). On the other hand, the deficiency of autophagy is linked to a corticosteroid-resistant asthma with neutrophilic inflammation, driven at least partially by IL-17—under such conditions, γδ T cells turn out to be the most important source of IL-17 (Suzuki et al. 2016).

In animal models of OVA-induced asthma, γδ T cell contribution to IL-17 production in BALF is related to the adjuvant used (Nakada et al. 2014). They seem to be of minor importance in the case of no adjuvant, of similar importance to Th17 in the case of aluminum-based adjuvant and of major importance in the case of complete Freud adjuvant (Nakada et al. 2014). The latter is especially not surprising as γδ T cells are well known for strong response to *Mycobacteria*, major component of complete Freud adjuvant (Zhao et al. 2018). By contrast, treatment with inactivated *M. phlei* caused symptom alleviation and near normalization otherwise increased percentages of Th-17-like γδ T and IL-23R^+^ γδ T in murine model of asthma (Ming et al. 2017). In the viral-mediated exacerbation of OVA-induced mice asthma, up to three fourths of total lung-infiltrating γδ T cells are Th17-like γδT, constituting 20–40% of total IL-17A^+^ cells in that compartment (Glanville et al. 2013).

Nevertheless, the data from murine model are contradictory to those in human asthmatic subjects, in whom no difference in Th17-like γδ T cells in either BALF, peripheral blood or induced sputum was observed when asthmatic patients were compared to healthy controls and between patients with asthma of different severity (Hinks et al. 2015; Zhao et al. 2011). Concluding, Th17-like γδ T cells were predominantly studied in animal model of asthma, in which they seem to be alleviating symptoms. Exact effect depends, to some extent, on stimulus used to establish the model. Data about Th17-like γδ T cells in human asthmatic subjects are scarce, but suggest a lesser role in asthma pathogenesis.

Apart from IL-17, Th17-like cells may also produce IL-22 (Zarobkiewicz et al. 2019c), a cytokine acting mostly on non-hematological cells, e.g., epithelial cells (Rutz et al. 2013). Under the majority of experimental settings, IL-22 was found to be protective in murine model of asthma; though in fungal asthma, it was found to be rather pathogenic (Hirose et al. 2018). Although γδ T cells can be the source of IL-22, they seem not to be an important one in fungal asthma (Reeder et al. 2018).

### Treg-Like γδT are of Minor Importance in Asthma

IL-10 is one of the major anti-inflammatory cytokines, during asthma, its concentration in BALF is significantly lowered; this probably predisposes to prolong inflammation (Trifunović et al. 2015). The depletion of γδ T cells in mice model of asthma exacerbation leads to significant decrease in BALF IL-10 level, which may suggest the important regulatory role of γδ T cells (Glanville et al. 2013). Moreover, a decrease in IL-10^+^ γδ T lymphocytes was observed in lungs of OVA-induced asthma in mice (Zhang et al. 2013a). The latter effect was significantly alleviated by the inhalation of inactivated *M. phlei* (Zhang et al. 2013a). On the other hand, according to Murdoch and Lloyd (2010), there are scarcely any IL-10^+^ γδ T cells in lungs of OVA-induced asthmatic mice. The opposite was reported in human asthmatic subjects—a significant up-regulation of IL-10^+^ γδ T cells was noted in induced sputum after short-term phytohemagglutinin stimulation (Hamzaoui et al. 2002). Data about Treg-like γδ T cells in asthma are scarce—while in animal models, they seem to be of some importance, in human asthma, Treg-like γδ T cells may be insignificant.

## Conclusions

Most of our current knowledge about γδ T cells in asthma stems from animal studies. Due to significant differences between γδ T cells in rodents and humans as well as between different experimental approaches used in those studies, those data cannot be easily extrapolated to human asthma. Unfortunately, γδ T cells in human asthma to date have not been extensively studied, still available results suggest their important role in pathogenesis of human asthma. More comprehensive studies (involving different functional subsets) on γδ T in human asthma are required to significantly advance our knowledge.

## Abbreviations

AHR: Airway hyperresponsiveness
APC: Antigen-presenting cell
BALF: Bronchoalveolar lavage fluid
CD: Cluster of differentiation
HMB-PP: (E)-4-hydroxy-3-methyl-but-2-enyl-pyrophosphate
HSP: Heat shock protein
IFN-γ: Interferon γ
IL: Interleukin
IPP: Isopentenyl pyrophosphate
OVA: Ovalbumin
RSV: Respiratory syncytial virus
TCR: T cell receptor
Tfh: T follicular helper cell
Th: T helper cell
Treg: Regulatory T cell

## Footnotes

1 Bim is a Bcl-2 family member with a pro-apoptotic function (Banjara et al. 2020).
